# Decoding the anticancer and biofilm-inhibiting efficacy of *Adansonia digitata* using experimental, AI-powered, and molecular modeling approaches

**DOI:** 10.3389/fmolb.2025.1666360

**Published:** 2025-10-29

**Authors:** Sangavi Pandi, Nachammai Kathiresan, Gowtham Kumar Subbaraj, Deepali Desai, Chitra Nagarajan, Langeswaran Kulanthaivel

**Affiliations:** ^1^ Center for Bioinformatics, Karpagam Academy of Higher Education, Coimbatore, Tamil Nadu, India; ^2^ Department of Microbiology, Karpagam Academy of Higher Education, Coimbatore, Tamil Nadu, India; ^3^ Department of Biotechnology, Alagappa University, Karaikudi, Tamil Nadu, India; ^4^ Faculty of Allied Health Sciences, Chettinad Hospital and Research Institute, Chettinad Academy of Research and Education (Deemed to be University), Kelambakkam, Tamil Nadu, India; ^5^ Department of Microbiology, Dr. D. Y. Patil Medical College Hospital and Research Centre, Dr. D. Y. Patil Vidyapeeth (Deemed to be University), Pimpri, India; ^6^ Department of Microbiology, Sri Venkateswara Dental College and Hospital (Affiliated to The TN Dr. MGR Medical University), Chennai, India; ^7^ Department of Biomedical Science, Alagappa University, Karaikudi, Tamil Nadu, India

**Keywords:** *Adansonia digitata*, MDA-MB-231, biofilm inhibition, AI-based docking, XP docking, molecular dynamics simulation, MMPBSA and PCA/FEL analysis

## Abstract

**Introduction:**

*Adansonia digitata*, commonly known as the Baobab tree, is a highly multifunctional species with significant cultural and economic value across various regions of Africa. This study aims to investigate the anticancer and cytotoxic properties of ethanol extract derived from *A. digitata* (ADEE) on MDA-MB-231 breast cancer cells, as well as its potential to inhibit biofilm formation.

**Methods:**

The study employs GNINA, a deep learning-based docking tool, to evaluate molecular interactions. This work integrates machine learning and molecular modeling methodologies, highlighting the potential of informatics-driven strategies to expedite the discovery of novel plant-based therapies.

**Results and Discussion:**

Fluorescence microscopy demonstrated that ADEE effectively inhibited biofilm formation and reduced cell viability at a concentration of 1.56 μg/mL. These findings suggest that ADEE disrupts quorum-sensing signaling pathways and compromises the structural integrity of the biofilm matrix. Further assessments of cytotoxicity revealed a dose-dependent reduction in cancer cell viability, highlighting the potent anticancer properties of ADEE. The study also confirmed the pro-apoptotic effects of ADEE through Hoechst and AO/EB staining techniques. Validation utilizing GNINA-based deep learning techniques demonstrated an enhanced binding affinity and pose stability of compounds derived from ADEE. Molecular dynamics simulations provided insights into the interactions of ADEE with pqsA and CK2, showing more favorable binding characteristics compared to the reference inhibitor. PCA/FEL analyses indicated stable conformations with significant interactions at critical residues. In summary, the phytocompounds identified in ADEE demonstrated enhanced binding affinity and structural stability, indicating promising therapeutic potential for targeting QS-regulated biofilm development and serving as potential anticancer agents.

## 1 Introduction

Contemporary research on drug development from medicinal plants utilizes a multifaceted strategy that combines botanical studies, phytochemical analysis, biochemical combinatorial chemistry, and bioassay-guided fractionation techniques (Chihomvu et al., 2024). Pharmaceuticals derived from these natural sources are effective against various debilitating diseases, including diabetes, cardiovascular diseases, and cancer. As the demand for safe and effective medications continues to grow, researchers are increasingly looking to nature for potential therapies for these chronic and often deadly illnesses (Katiyar et al., 2012). However, significant challenges exist in this field, particularly in scaling up and commercializing active compounds. As a result, only about one in every thousand lead molecules are successfully developed into a medicine. Medicinal plants are key natural resources for managing chronic illnesses. The types of medicinal plants used to treat similar conditions can vary by country (Sofowora et al., 2013). In certain regions, these plants are valued for their historical uses and serve as affordable alternatives for treating various health issues. However, further ethnobotanical research is needed to quantitatively assess the use of medicinal plants, their toxicological effects, and their efficacy when used as a sole treatment for various illnesses (Obakiro et al., 2023).

One notable example is the African baobab tree, scientifically known as *Adansonia digitata* L. This tree is one of the most important wild foods plants and holds significant ecological and social importance in much of tropical Africa (Gebauer et al., 2016). Within the genus *Adansonia*, there are nine species of baobab found worldwide. Various solvent extracts from the tree have shown medicinal properties. Studies indicate that extracts from the bark of *A. digitata* can effectively treat inflammation, diabetes mellitus, and microbial infections (Sangavi et al., 2024a; Offiah and Falade, 2023). Additionally, several flavonoids present in the tree’s bark are utilized as remedies for fever and serve as alternatives to cinchona bark in Europe, where some of these compounds are marketed under the name “cortex cael cedra.” The baobab tree is a notable species native to the thorn woodlands of the African savannahs (Assogba et al., 2021). This tree is highly versatile, with edible parts including seeds, leaves, roots, flowers, fruit pulp, and bark. The fruit pulp is an excellent appetizer or beverage, rich in vitamin C, proteins, calcium, phosphate, potassium, carbohydrates, fiber, and lipids. Additionally, the seeds contain significant amounts of sodium, phosphorus, zinc, iron, magnesium, manganese, lysine, thiamine, and calcium. In traditional medicine, *A. digitata* offers various nutritional benefits and has therapeutic potential for treating conditions such as inflammation, malaria, diarrhea, anemia, tuberculosis, dysentery, and toothache (Rahul et al., 2015). The extract from the stem bark showed significant anti-parasitic effects in mice infected with *Plasmodium berghei*. It increased packed cell volume and reduced parasitemia, along with decreasing tissue peroxidation and boosting antioxidant levels. Dispensing the extract after infection lowered inflammatory markers and severity of illness, indicating its potential as an effective malaria treatment. The baobab tree, revered across Africa for its tremendous medicinal and nutritional value, has been an integral part of traditional medicine and local diets for centuries. Every part of the baobab seeds, leaves, roots, flowers, fruit pulp, and bark is edible and highly versatile. The leaves are often used to prepare soups, while the seeds can act as a thickening agent or flavoring ingredient, or they can be roasted and eaten as snacks. Additionally, baobab seeds can be fermented to enhance their flavor. Beyond its culinary uses, the baobab is celebrated for its antioxidant and anti-inflammatory properties. Traditionally, it has been employed to treat ailments such as diarrhea, malaria, and various microbial infections (Malabadi et al., 2021). An alkaloid called “adansonine,” isolated from the stem bark, has been linked to antimalarial and antidepressant effects (Shehu et al., 2017). Additionally, Norcinnamolaurine, also derived from the bark, demonstrates a strong binding affinity for the protein AURKA, suggesting significant bioactivity and stability. Studies on its vibrational and Frontier Molecular Orbital (FMO) properties highlight its molecular behavior, with factors like energy gap and solvation effects enhancing its potential for various applications (Sangavi et al., 2024b). The baobab tree holds a unique place in African culture and is a symbol of health and resilience in many communities.

Triple-negative breast cancer (TNBC) and biofilm-associated infections illustrate significant global health challenges. TNBC constitutes 15%–20% of all breast cancer cases and is associated with a poor prognosis due to the lack of targeted therapies and the prevalence of chemoresistance. Likewise, microbial biofilms are involved in over 80% of chronic infections, rendering conventional antibiotics inefficient because of their limited penetration and the effect of resistance mechanisms. Consequently, there is an acute necessity to investigate innovative treatment approaches that can address both cancer progression and biofilm formation.

In our prior study (Sangavi et al., 2024a), we demonstrated that *A. digitata* ethanol extract (ADEE) exhibited significant antioxidant and antibiofilm properties, as evidenced by *in vitro* evaluations utilizing the DPPH and crystal violet biofilm inhibition methods. Additionally, molecular docking studies were conducted to identify key phytoconstituents with strong binding affinities to receptors implicated in quorum sensing and the regulation of oxidative stress. However, the molecular association between ADEE’s antioxidant efficiency, antibiofilm efficacy, and cytotoxic effects on cancer cells remains insufficiently understood.

Based on these findings, the present study aims to further evaluate and expand upon this understanding by performing microscopic assessments of biofilm disintegration and assessing the cytotoxicity of ADEE against MDA-MB-231 cells. Moreover, employing the lead compounds identified in our earlier docking investigations, we implemented molecular dynamics simulations on the quorum-sensing protein pqsA and the antioxidant-related Protein Kinase CK2. This comprehensive approach seeks to elucidate ADEE’s dual therapeutic role in modulating both microbial biofilms and redox-dependent cancer pathways. The dual functionality of ADEE in disrupting biofilms and inducing apoptosis in cancer cells may be partially attributed to its antioxidant properties. This study integrates both *in vitro* and *in silico* techniques to enhance the understanding of ADEE’s dual therapeutic effects in combating microbial biofilms and modifying redox-sensitive cancer pathways. The findings may contribute to the development of natural product-based therapies for biofilm-associated infections and aggressive malignancies such as TNBC.

## 2 Methodology

### 2.1 Disintegration of mature *Pseudomonas aeruginosa* biofilm

Overnight cultures of *Pseudomonas aeruginosa* were inoculated into 1 mL of fresh LB broth in a 24-well microtiter plate. Glass slides (1 cm × 1 cm) were added to each well to provide a surface for biofilm formation (Mustha et al., 2011). The plates were incubated at 37 °C for 18 h, either with or without 1.56 μg/mL of ADEE. After incubation, the planktonic cells and spent media were removed. The glass slides with adherent biofilms were gently rinsed twice with deionized water and allowed to air dry (Rashiya et al., 2021). The biofilms were stained with 0.1% (w/v) acridine orange for microscopic analysis. The excess stain was then removed by rinsing the slides twice with deionized water, followed by air drying. Stained biofilms were visualized using a Nikon Eclipse Ti microscope.

### 2.2 Cytotoxicity assay

To evaluate the cytotoxic effects of ADEE on MDA-MB-231 TNBC cells, a dose-dependent MTT assay was performed over a 24-h interval. The MDA-MB-231 cells were cultured in DMEM containing 10% fetal bovine serum and 1% penicillin-streptomycin under standardized conditions (37 °C, 5% CO_2_) (Vinita et al., 2023). ADEE was prepared in DMSO, diluted with DMEM, and applied to the cells at varying concentrations, while control wells received only DMSO. The cells were seeded in a 96-well plate at a density of 5 × 10^3^ cells per well and were allowed to adhere overnight before the treatment with ADEE. Following 24 h, the cells were aspirated, and 20 µL of MTT solution (5 mg/mL in PBS) was added to each well. The plates underwent incubation at 37 °C for 4 h to facilitate the formation of formazan crystals. A Bio-Rad microtiter plate reader was used to measure absorbance at 595 nm after the MTT solution was removed (Mali et al., 2017). Subsequently, 100 µL of DMSO was added to each well to dissolve the crystals. The data were statistically analyzed to assess the significance of the variance between the treated and control groups, which revealed a dose-dependent cytotoxic effect of ADEE on MDA-MB-231 cells. Cell viability was calculated as a percentage of the control absorbance readings using the following formula,

$$Cell\;Viability\;\left(\%\right)=\left(\frac{Absorbance\;of\;Control\;cells}{Absorbance\;of\;Treated\;cells}\right)\times 100$$

### 2.3 Apoptotic efficacy of ADEE

Acridine orange/ethidium bromide (AO/EtBr) double staining and Hoechst 33342 staining were employed to detect apoptotic changes. MDA-MB-231 cells were stained with acridine orange/ethidium bromide and Hoechst 33342 for 15 min in both the control and ADEE-treated cells (9.17 μg/mL). The inhibitory activity of ADEE was assessed on the labeled cells using a fluorescence microscope (Kumar et al., 2024).

### 2.4 AI-based docking validation of pqsA and 2OXX complexes

In our previous study, we analyzed the binding efficacy of phytocompounds extracted from ADEE with pqsA and Protein Kinase CK2 (Sangavi et al., 2024a). In order to validate the molecular docking data obtained from our prior investigation utilizing Schrödinger software, we employed GNINA, an artificial intelligence-integrated docking tool accessible through the Neurosnap platform (Neurosnap Inc, 2022). GNINA 1.0 utilizes convolutional neural networks to assess protein-ligand poses and predict binding affinity and pose quality (McNutt et al., 2021). The docking procedure was conducted using GNINA’s default docking method, which is rooted in the AutoDock Vina 1.2.0 engine (Eberhardt et al., 2021) and enhances the efficacy of the original Vina scoring and sampling techniques (Trott and Olson, 2010). Docking procedures were executed by uploading the generated protein and ligand files to the GNINA interface within the Neurosnap platform. The configurational parameters included cnn_scoring = cnnscore and a setting of num_modes equal to 9, which resulted in multiple binding conformations. The selection of the CNN score and the number of modes was reported by previous GNINA benchmarking studies, which support these parameters to ensure accurate docking predictions while maintaining computational efficiency. The output metrics comprised the CNN Pose Score, indicating pose confidence, and the CNN Affinity, representing the anticipated binding free energy. The top-ranked binding conformations were subsequently compared across various compounds for both targets to facilitate further molecular dynamics simulations.

### 2.5 Molecular dynamics simulation

The top phytocompounds demonstrated a better docking score and significant interactions with key residues, which facilitate biofilm formation by pqsA and the Antioxidant enzyme CK2. In this study, we examine the structural stability, residual mobility, and interaction affinity of the docked complexes using Molecular Dynamics Simulation (MDS). Additionally, we have Tetracycline as the reference inhibitor at the putative site of pqsA for validation. MDS was conducted on the top-docked complexes to evaluate the stability of the protein-ligand interactions. We utilized GROMACS 2022-1 to perform the molecular dynamics simulations, employing the CHARMM36 force field (Huang et al., 2017) and the TIP3P water model to solvate the complexes. The ligand topology was constructed using the Param-Chem server in conjunction with the CHARMM General Force Field (CGenFF). Additionally, the compounds were placed in a cubic box with a 1.0 nm dimension and neutralized with sodium and chloride ions. The system underwent energy minimization over 10,000 steps using the steepest descent method, with the positions of the complexes constrained. The bond lengths were constrained using the LINCS (Linear Constraint Solver) method, while long-range electrostatic interactions were calculated using the PME (Particle Mesh Ewald) approach. The system underwent a 100-ps NVT ensemble simulation at 300 K, conducted with a specific number of particles, volume, and temperature. This was followed by a 100 ps NPT equilibration run at 300 K and 1 bar of pressure. During the equilibration, both the target temperature and the duration were set to 300 K and 0.1 ps, respectively. The pressure was maintained at 1 bar using the Parrinello-Rahman barostat algorithm, which featured a time constant of 1 ps (El Rhabori et al., 2024). Subsequently, the complexes were subjected to an MD run for 300 ns; further, the trajectory was analyzed and visualized through GROMACS and xmgrace.

### 2.6 MMPBSA

To calculate the binding energy of biomolecular complexes, the MMPBSA method is widely used. Its integration with molecular dynamics simulations (MDS) provides a useful approach for studying biomolecular interactions (Al-Khafaji and Tok, 2020). The change in binding free energy is determined by comparing various energy components, including van der Waals, electrostatic, and solvation energies, using MMPBSA. In this study, we employed the “gmx_mmpbsa” plugin for GROMACS to calculate binding free energy (Val et al., 2021). This calculation was based on stable trajectories obtained from a 300 ns MDS run.

### 2.7 Essential dynamics

Principal component analysis (PCA) was employed to investigate the underlying dynamics of protein-ligand interactions. PCA is a multivariate statistical method that begins with a covariance matrix and linearly reduces the data to uncover the most significant features or movements within complex trajectories (Singh et al., 2024). The covariance matrix in PCA is used to determine the eigenvectors and eigenvalues. The motion corresponding to an eigenvector increases as its associated eigenvalue rises. This approach enables the exploration of the complexity of collective motion, which is linked to system stability and protein function, by varying parameters and simplifying the motion (Aarthy and Singh, 2021). PCA calculations were performed using the built-in functions g_covar and g_anaeig from the GROMACS software package.

## 3 Results

### 3.1 Microscopic analysis of ADEE’s effect on *Pseudomonas aeruginosa*

**FIGURE 1:**
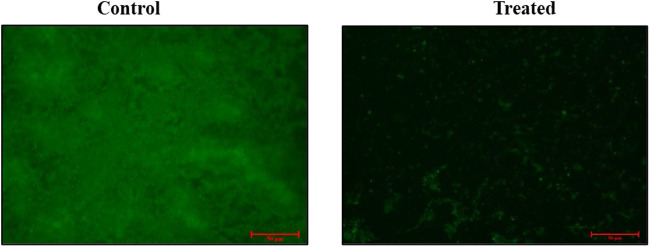
Fluorescence microscopy study demonstrating the inhibitory effect of *Adansonia digitata* bark ethanolic extract (ADEE) on biofilm formation by *Pseudomonas aeruginosa*.

*Pseudomonas aeruginosa* is a notorious pathogen known for its ability to form biofilms, a process often regulated by QS. Biofilm formation significantly enhances bacterial antibiotic resistance and contributes to severe systemic infections. In this study, fluorescence microscopy results demonstrated the biofilm inhibition activity of ADEE against *P. aeruginosa*. Our previous research reported the biofilm-inhibiting efficacy of ADEE using the Crystal Violet method at different concentrations. This current study focused on the inhibitory efficacy of ADEE in a dose-dependent manner, employing fluorescence microscopy at a concentration of 1.56 μg/mL. The control images displayed the presence of mature biofilms with viable cells, observed as intense green fluorescence. This could be due to inhibition of the QS signal or disruption of the biofilm matrix. In contrast, the ADEE-treated samples showed a reduced number of viable cells and disrupted biofilm formation, confirmed by a decrease in fluorescence intensity. These findings are illustrated in Figure 1, and the results suggest that ADEE is effective in impairing biofilm formation by *P. aeruginosa*.

### 3.2 Cytotoxicity assay

**FIGURE 2:**
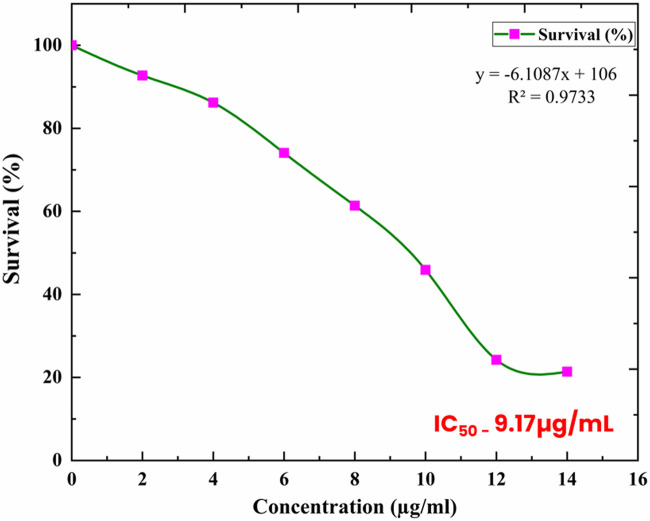
Dose-dependent cytotoxic effect of ADEE on MDA-MB-231 cells as determined by the MTT assay.

The findings of this study demonstrate that the bark extract of ADEE shows dose-dependent toxicity toward MDA-MB-231 breast cancer cells, as determined by the MTT assay. The graph (Figure 2) presents a distinct trend, indicating that cell viability diminishes as the concentration of ADEE rises. This toxic effect is dependent on concentration, backed by a high correlation coefficient (R^2^ = 0.9733) derived from the equation (y = −6.1087x + 106). The R^2^ value explains 97.33% of the variability in cell survival, highlighting a strong, linear connection between ADEE exposure and cell viability. The IC_50_ value of 9.17 μg/mL represents a significant finding in the study. This value denotes the concentration of ADEE necessary to achieve a 50% reduction in the viability of MDA-MB-231 cells, highlighting the compound’s potent anticancer properties even at low dosages. At the lowest concentration tested, 2 μg/mL, cell survival was recorded at 92.72%, indicating minimal cytotoxic effects. An increase in concentration to 6 μg/mL resulted in a notable decline in cell survival to 74.09%, demonstrating enhanced cytotoxic activity. At a concentration of 10 μg/mL, the viability of the cells decreased further to 45.91%, and at the highest dosage assessed, 14 μg/mL the survival rate decreased dramatically to 21.37%.

The findings underscore the considerable anticancer potential of ADEE, demonstrating its capability to significantly diminish the viability of MDA-MB-231 breast cancer cells at relatively low concentrations. The IC_50_ value of 9.17 μg/mL indicates that ADEE can achieve a 50% reduction in cell survival without necessitating high dosages, a desirable characteristic for prospective therapeutic agents. This dose-response relationship suggests that ADEE warrants further investigation as a natural anticancer agent, particularly in targeting aggressive breast cancer cell lines such as MDA-MB-231.

### 3.3 Apoptotic efficacy of ADEE against MDA-MB-231

**FIGURE 3:**
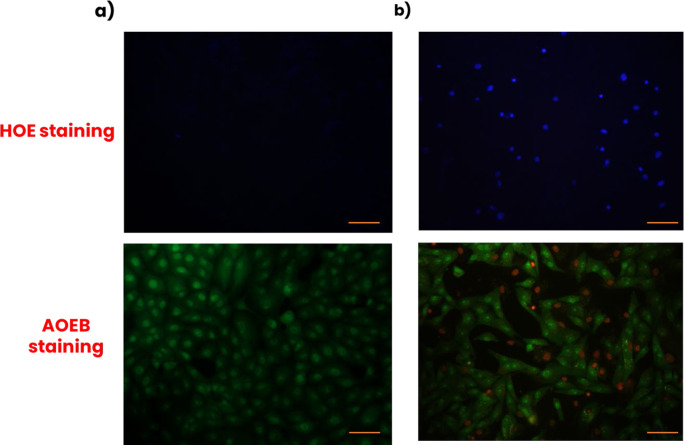
Apoptosis-inducing effect of ADEE in MDA-MB-231 breast cancer cells envisioned by Hoechst 33342 (HOE) and Acridine Orange/Ethidium Bromide (AO/EB) staining. **(a)** Control. **(b)** Treated with ADEE (IC_50_-9.17 μg/mL).

The apoptotic effects of ADEE on MDA-MB-231 breast cancer cells were evaluated using Hoechst (HOE) and Acridine Orange/Ethidium Bromide (AO/EB) staining techniques (Figure 3). In the control group (Figure 3a), HOE staining showed faint and uniform blue fluorescence, indicating healthy nuclei without chromatin condensation or nuclear fragmentation. Meanwhile, AO/EB staining exhibited green fluorescence, signifying live cells with intact membranes. In contrast, cells treated with ADEE at its IC_50_ concentration of 9.17 μg/mL (Figure 3b) displayed intense blue fluorescence under HOE staining, which suggested chromatin condensation and nuclear disintegration, both characteristic markers of cell death. The AO/EB labeling of these treated cells revealed a mix of green and orange/red fluorescence. The green fluorescence indicated early apoptosis, while the orange/red fluorescence pointed to late apoptosis or necrosis due to compromised membrane integrity. These findings indicate that ADEE induces apoptosis in MDA-MB-231 cells, highlighting its potential as a therapeutic agent for triple-negative breast cancer.

### 3.4 The binding affinity of pqsA with tetracycline (reference inhibitor)

**FIGURE 4:**
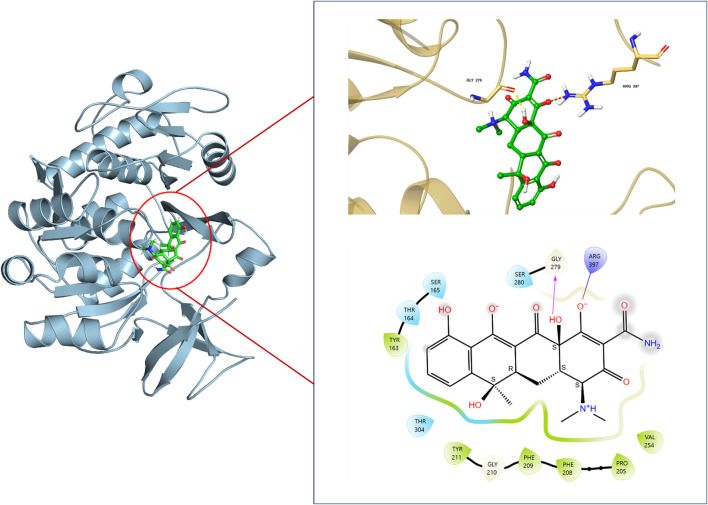
Binding interaction of Tetracycline (Reference inhibitor) with the binding pocket of pqsA protein, showing docking pose, interacting residues, and 2D & 3D interaction pattern of Protein_Ligand complex.

We have used Tetracycline as the standard to compare the efficacy of ADEE in inhibiting biofilm formation. In this study, we docked Tetracycline at the putative site of pqsA, where it demonstrated a docking score of −7.243 kcal/mol. This interaction included one hydrogen bond with Gly279 and a salt bridge with Arg397 (Figure 4). In comparison to Tetracycline, the lead phytocompounds we identified exhibited better docking scores and significant interactions with crucial residues, as detailed in our previous study (Sangavi et al., 2024a). As a continuation of this work, we have conducted further analyses to elucidate the binding efficacy and structural stability of the complexes via MD simulation.

### 3.5 GNINA-based docking validation

#### 3.5.1 Docking interpretation of pqsA complexes

**FIGURE 5:**
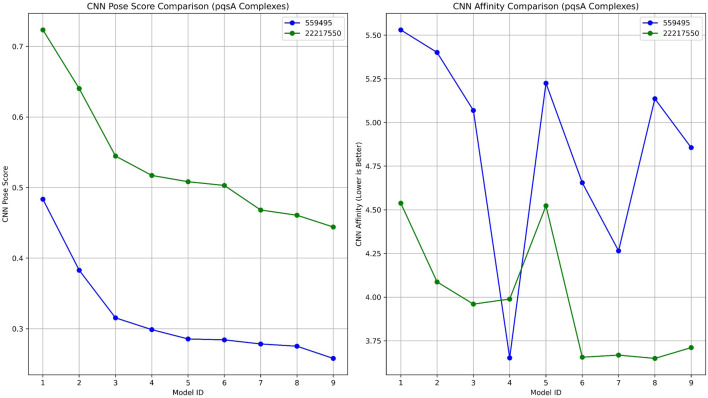
A comparative analysis of GNINA regarding pqsA binding with compounds 559495 and 22217550, illustrating the CNN Pose Score and the CNN-predicted binding affinity.

To validate the ligand-binding conformations obtained from our prior docking investigation utilizing Schrödinger, we rescored and evaluated the top-ranked complexes pqsA_22217550 and pqsA_559495 using GNINA (Figure 5; Table 1). Among the two compounds, CID_22217550 demonstrated a significantly higher CNN Pose Score of 0.7234, indicating a strong level of confidence in its predicted binding conformation. Moreover, it received a favorable CNN Affinity score of 4.538, suggesting a high probability of advantageous interaction with the binding site. This assessment aligns closely with its earlier Schrödinger Glide score of −6.743 kcal/mol, thereby reinforcing the validity of its docking position from both traditional and AI perspectives. In addition, it exhibited a low intramolecular strain of −0.34 kcal/mol, which contributes to its structural stability within the binding site.

**TABLE 1:** Docking results for pqsA complexes utilizing GNINA.

Model ID	Affinity (kcal/mol)	Intramolecular energy (kcal/mol)	CNN pose score	CNN affinity	CNN VS	CNN variance
a) PqsA_22217550						
1	−6.53	−0.34	0.723	4.538	3.282	0.125
2	−5.61	−0.52	0.640	4.087	2.616	0.316
3	−6.53	−0.54	0.544	3.96	2.156	0.387
4	−4.42	−0.52	0.517	3.989	2.063	0.072
5	−4.75	−0.55	0.508	4.523	2.299	0.077
6	−6.21	−0.35	0.503	3.656	1.839	0.715
7	−4.86	−0.37	0.468	3.668	1.717	0.473
8	−4.95	−0.53	0.460	3.649	1.681	0.602
9	−5.24	−0.53	0.444	3.711	1.648	0.356
b) pqsA_559495						
1	−7.18	−0.37	0.483	5.53	2.673	0.734
2	−6.93	−0.01	0.382	5.401	2.067	0.162
3	−6.81	0.79	0.315	5.069	1.598	2.357
4	−4.56	−0.58	0.298	3.652	1.090	1.082
5	−6.78	1.2	0.285	5.225	1.491	0.637
6	−4.93	−0.45	0.284	4.655	1.322	0.447
7	−5.74	0.45	0.278	4.265	1.187	0.701
8	−6.88	−0.27	0.275	5.136	1.413	0.270
9	−4.67	−0.76	0.257	4.856	1.252	0.140

In contrast, compound 559495 presented a marginally higher docking score in Schrödinger (−7.053 kcal/mol), yet showed a lower CNN Pose Score of 0.4835 and a higher CNN Affinity score of 5.530. These results imply a more ambiguous or less favorable binding orientation as suggested by the AI model. The internal energy of this compound was evaluated at −0.37 kcal/mol, which remains within an acceptable range. In conclusion, the analysis based on GNINA demonstrates CID_22217550 as the most promising candidate, supported consistently by both conventional docking scores and machine learning validation.

#### 3.5.2 Interpretation of 2OXX complexes

**FIGURE 6:**
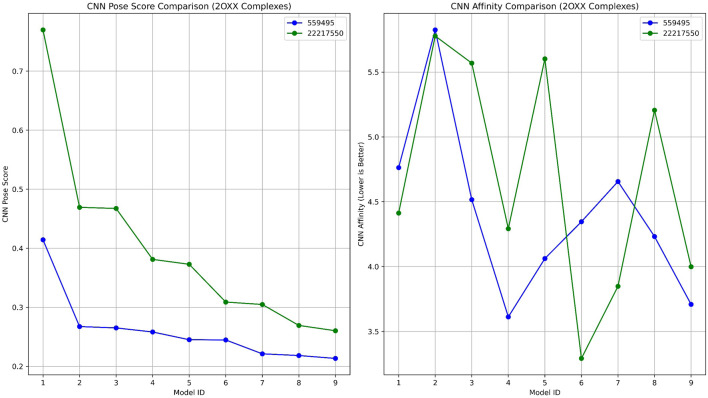
A comparative analysis of GNINA validation 2OXX binding with compounds 559495 and 22217550, illustrating the CNN Pose Score and the predicted binding affinity from the CNN model.

Lead complexes 2OXX_559495 and 2OXX_22217550 were reassessed using AI-based rescoring with GNINA (Figure 6; Table 2). The docking scores provided by Schrödinger showed a slight preference for compound 559495, which acquired a score of −7.573 kcal/mol, compared to −7.462 kcal/mol for CID_22217550. This suggests that the binding potentials of the two compounds are quite similar. Yet, the evaluation conducted with GNINA provided a more in-depth understanding of pose quality and projected binding affinity from a machine learning perspective. Among the top-ranked models, compound CID_22217550 consistently achieved higher CNN Pose Scores than compound 559495, with a peak score exceeding 0.78. This outcome implies that the AI displays considerable confidence in the predicted ligand configuration for CID_22217550. In contrast, compound 559495 yielded lower CNN Pose Scores across the majority of poses, remaining below 0.42, which signifies a diminished reliability regarding pose quality.

**TABLE 2:** Results from deep learning docking studies of CK2 complexes using GNINA.

Model ID	Affinity (kcal/mol)	Intramolecular energy (kcal/mol)	CNN pose score	CNN affinity	CNN VS	CNN variance
a) 2OXX_22217550						
1	−4.94	−0.34	0.769	4.413	3.397	0.035
2	−9.72	0.12	0.469	5.778	2.711	0.003
3	−10	0.19	0.467	5.569	2.603	0.176
4	−5.17	−0.5	0.381	4.292	1.635	0.225
5	−9.28	−0.35	0.372	5.602	2.088	0.082
6	−6.67	−0.34	0.308	3.292	1.016	0.799
7	−5.25	−0.46	0.304	3.848	1.172	0.285
8	−9.04	−0.46	0.269	5.206	1.401	0.086
9	−4.99	−0.52	0.260	3.999	1.040	0.297
b) 2OXX_559495						
1	−4.95	−0.51	0.414	4.763	1.973	0.113
2	−8.16	0.67	0.267	5.825	1.557	0.033
3	−4.81	0.32	0.265	4.516	1.196	0.060
4	−5.46	−0.35	0.258	3.612	0.932	1.338
5	−5.42	−0.2	0.245	4.062	0.995	0.609
6	−5.1	−0.82	0.244	4.346	1.063	0.276
7	−4.41	0.29	0.221	4.656	1.029	0.230
8	−5.31	−0.17	0.218	4.232	0.923	0.386
9	−4.6	−0.38	0.213	3.709	0.791	1.142

Moreover, the CNN Affinity plots indicated that compound CID_22217550 presented lower projected affinities across multiple models compared to compound CID_559495. The latter exhibited greater variability and overall higher CNN Affinity scores, reaching up to 5.8 kcal/mol. These findings suggest that, despite the initial preference indicated by Schrödinger docking for compound 559495, GNINA identifies compound CID_22217550 as the superior binder in terms of both conformation and binding probability, as assessed by deep learning techniques.

### 3.6 Molecular dynamics simulation of pqsA with the phytocompounds

#### 3.6.1 Structural stability of pqsA complexes

**FIGURE 7:**
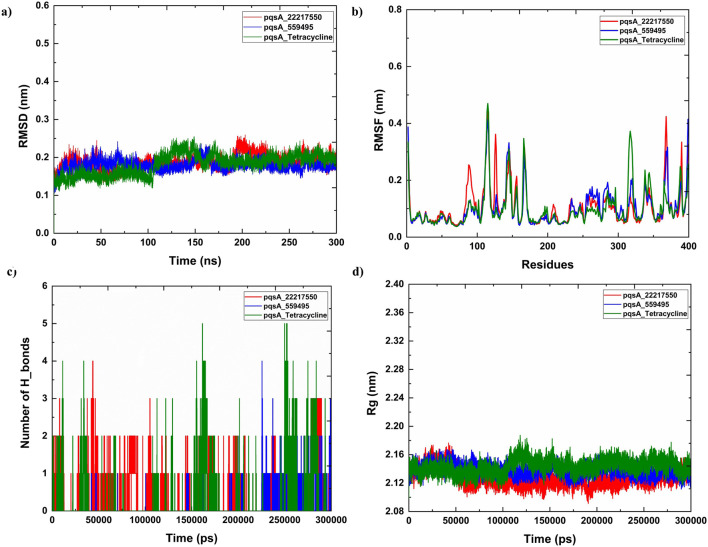
The analysis of molecular dynamics (MD) simulations for pqsA complexes investigates the interactions with two phytocompounds (CID_22217550 and CID_559495) as well as tetracycline. **(a)** Root Mean Square Deviation (RMSD) plots, which indicate the structural stability of the complexes; **(b)** Root Mean Square Fluctuation (RMSF) values, which reveal the flexibility of individual residues; **(c)** hydrogen bond occupancy; and **(d)** the radius of gyration (Rg), which aids in representing the overall compactness of the protein.

Molecular dynamics simulations were performed to evaluate the structural stability and residual mobility of the protein-ligand complexes for 300 ns (Figure 7). Further, the trajectories were used to determine the stability of the complexes through the Root Mean Square Deviation (RMSD) (Figure 7a). Throughout the 300 ns simulation, the average RMSD values of pqsA complexes were observed to be within the range of less than 0.2 nm. The reference inhibitor Tetracycline bound to pqsA exhibited an average RMSD value of 0.19 nm, with a minimal deviation of 0.24 nm, and was found to be stable throughout the simulation. pqsA_559495 exhibited a lower average RMSD value of 0.178 nm with an SD of 0.01 nm. The complex remained stable throughout the simulation without any major deviations, indicating its structural stability. The observed lesser deviation of the minimum and maximum RMSD ranges was noted at 0.00049 nm and 0.24 nm, emphasizing the consistent stability observed throughout the simulation. The pqsA_22217550 complex exhibited an average RMSD value of 0.18 nm (SD = 0.017 nm), slightly higher than the pqsA_559495 complex. This complex demonstrated comparatively narrow minimum and maximum RMSD values of 0.00050 nm and 0.238 nm, respectively. These findings suggest that the complex is stable, showing minimal deviation. RMSD analysis indicated that pqsA exhibited considerable stability when interacting with the ligands, with tetracycline demonstrating superior structural stability compared to the others. Additionally, the lead complexes (pqsA_559495 and pqsA_22217550) obtained were relatively better than the reference inhibitor, as they had minimal deviation and greater structural stability throughout the simulation period.

#### 3.6.2 Residual mobility analysis

Root Mean Square Fluctuation (RMSF) was used to analyze the per-residue flexibility of the protein-ligand complexes. This study emphasizes the structural flexibility of pqsA complexes (CID_559495, CID_22217550, and Tetracycline) (Figure 7b). Notably, pqsA_559495 and pqsA_22217550 exhibited lesser deviation, observed at 0.23 nm, while the overall RMSF range was observed to be 0.5 nm. The complexes demonstrated less fluctuation in the binding regions, suggesting stabilization of the overall complex. The pqsA_559495 complex exhibits lesser fluctuations, with an average RMSF of 0.16 nm. Within the binding region, the residues demonstrate minimal fluctuation rates, specifically Glu305 at 0.09 nm, Gly302 at 0.09 nm, Thr380 at 0.08 nm, and Asp382 at 0.10 nm. The pqsA_22217550 complex demonstrated a reduced overall fluctuation rate, with an average RMSF of 0.17 nm, indicative of decreased flexibility throughout the protein structure. Notably, critical residues within the binding region, specifically Thr87 (0.19 nm) and Gly210 (0.10 nm), exhibited remarkably low variation values. These diminished fluctuations reflect enhanced local stability at the ligand-binding interface, thereby reinforcing the structural integrity and favorable interaction pattern of the pqsA_22217550 complex.

The complexes exhibited a lower standard deviation (CID_559495 - 0.06 nm, CID_22217550 - 0.07 nm, and Tetracycline - 0.07 nm). The maximum and minimum RMSF values were found to be better in the complexes, suggesting that ligand binding enhances structural stability and reduces the fluctuations of the residues. Overall, RMSF analysis revealed that the phytocompounds demonstrated relatively better stability and less fluctuation compared to the reference inhibitor.

#### 3.6.3 Hydrogen bond interaction

Analyzing hydrogen bond interactions provides insights into the binding affinity of protein-ligand complexes, which are non-covalent interactions that significantly contribute to the stability of the complex (Figure 7c). pqsA_559495 complex has shown 2 to 6 hydrogen bonds, which decipher the binding affinity and stability between the target and the ligand. The pqsA_22217550 complex demonstrated consistent hydrogen bond interactions throughout the simulation. Tetracycline exhibited the strongest hydrogen bond interaction with pqsA, which enhances binding affinity and stability. A higher number of interactions indicates greater binding affinity and stability of the complex. In this case, the phytocompounds also showed better hydrogen bond interactions with pqsA, contributing to overall structural stability throughout the simulation. Moreover, the structural compactness of the complex was analysed through the Radius of Gyration (Rg), which was depicted in Figure 7d. The analysis of the Rg elucidates the structural compactness and stability of the complex. Throughout the simulation phase, the resultant complex has demonstrated consistent stability. The average Rg values for the pqsA complexes varied from 2.13 nm to 2.18 nm throughout the simulation period. This limited range of Rg values indicates that the complexes remained compact and well-structured during the simulations, with no significant unfolding or expansion occurring. The consistency of these Rg values reinforces the overall structural stability of the protein-ligand complexes, suggesting that ligand binding does not induce substantial conformational alterations in the pqsA protein. Consequently, the MD simulation indicates that the resultant structures exhibit minimal deviation and fluctuation, accompanied by significant compactness. This is further corroborated by the observed structural stability of the complex, which is evidenced by hydrogen bond interactions. Further, the structural stability and folding of the docked complexes was evaluated by Solvent Accessible Surface Are (SASA) which is illustrated in Supplementary Figure S1a.

### 3.7 MMPBSA

**FIGURE 8:**
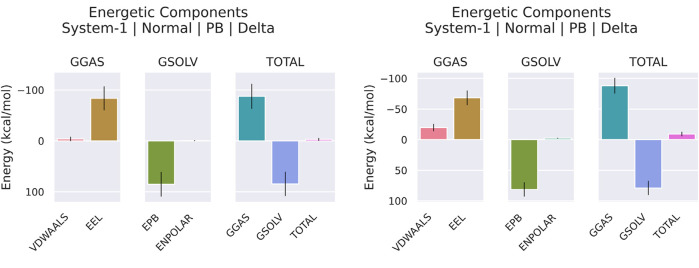
Binding free energy analysis of pqsA complexes with the phytocompounds (CID_559495 and CID_22217550) was calculated using MM/PBSA, highlighting the comparative binding affinities and stability of ligand–protein interactions.

The MMPBSA analysis demonstrated significant variations in the binding properties of the two pqsA complexes, which is tabulated in Table 3; Figure 8. For PqsA_559495 (Table 3), the total binding free energy is −3.01 ± 2.66 kcal/mol. Strong electrostatic interactions (EEL = −83.65 ± 23.52 kcal/mol) play a key role in achieving this low energy; however, this is counterbalanced by a considerable polar solvation penalty (EPB = 85.15 ± 24.18 kcal/mol). Consequently, the contributions from van der Waals interactions are relatively low (VDWAALS = −3.92 ± 4.33 kcal/mol), and nonpolar solvation effects are negligible (ENPOLAR = −0.59 ± 0.67 kcal/mol). pqsA_22217550 (Table 3) exhibits higher binding stability, with a total binding free energy of −9.12 ± 3.66 kcal/mol. This stability is primarily due to enhanced van der Waals interactions (VDWAALS = −19.72 ± 5.99 kcal/mol) and a somewhat reduced polar solvation penalty (EPB = 81.34 ± 11.59 kcal/mol). Although the electrostatic contribution (EEL = −68.34 ± 12.00 kcal/mol) is less favorable compared to pqsA_559495, the combination of stronger nonpolar interactions and lower solvation penalties results in more stable binding for pqsA_22217550. Furthermore, the reduced fluctuation in critical energy parameters for pqsA_22217550 indicates more consistent interactions across simulation frames, highlighting its improved stability over pqsA_559495.

**TABLE 3:** MMPBSA analysis of the pqsA complexes.

Compounds	Frames	VDWAALS	EEL	EPB	ENPOLAR	GGAS	GSOLV	Total
pqsA_559495	Average	−3.92	−83.65	85.15	−0.59	−87.57	84.56	−3.01
	SD	4.33	23.52	24.18	0.67	24.6	23.95	2.66
	SEM	0.19	1.05	1.08	0.03	1.1	1.07	0.12
pqsA_22217550	Average	−19.72	−68.34	81.34	−2.41	−88.05	78.93	−9.12
	SD	5.99	12	11.59	0.5	12.72	11.54	3.66
	SEM	0.19	0.38	0.37	0.02	0.4	0.36	0.12

### 3.8 Essential dynamics

**FIGURE 9:**
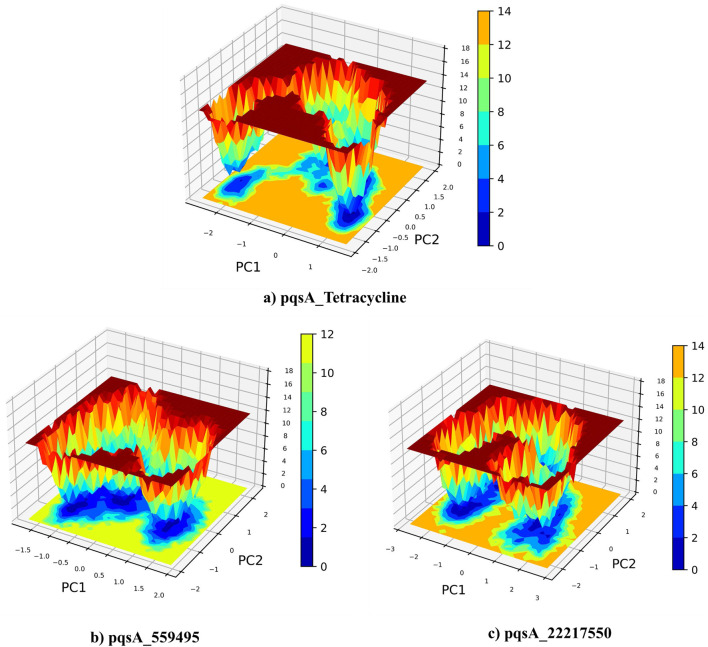
Principal component analysis (PCA) and the analysis of the free energy landscape (FEL) for pqsA complexes: Tetracycline **(a)**, CID_559495 **(b)**, and CID_22217550 **(c)**, illustrating the predominant conformational motions and energy minima that contribute to the stability of these complexes.

**FIGURE 10:**
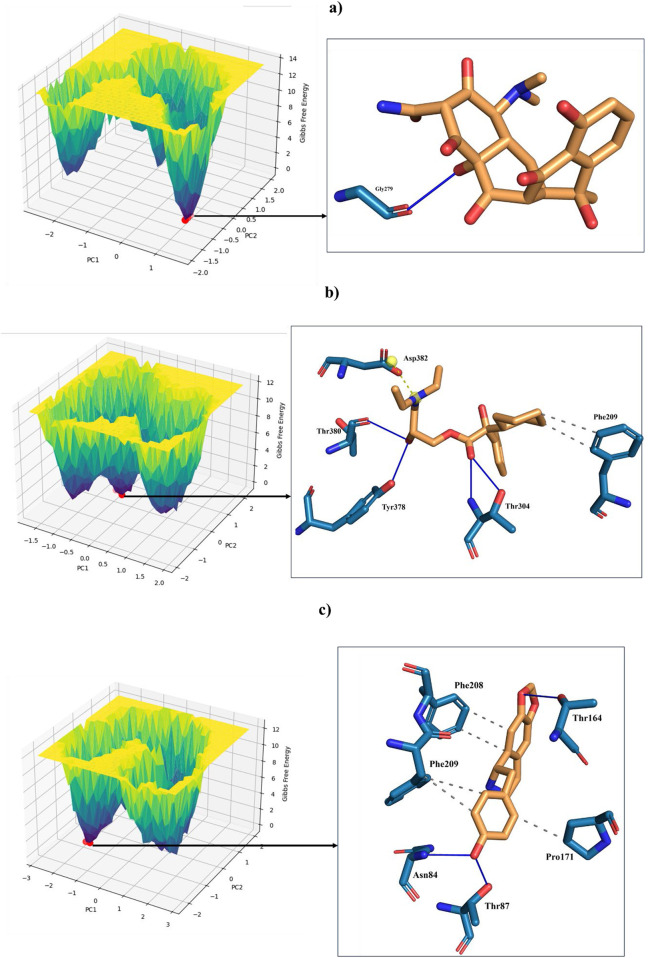
FEL (Left) derived from PCA of pqsA complexes, highlighting key Protein_Ligand interactions (right) at the global minimum energy state. **(a)** pqsA_Tetracycline. **(b)** pqsA_559495. **(c)** pqsA_22217550.

Principal Component Analysis (PCA) and Free Energy Landscape (FEL) analysis were conducted to identify the most stable conformations and detect changes in the target molecule from the MD trajectories. In this study, we performed PCA/FEL analysis on pqsA complexes, highlighting the system’s most significant transitions and the most favorable states. Figures 9, 10 display the PC1 and PC2 components of the complexes, illustrating the trajectory’s primary motions of pqsA complexes (Figure 9a). The pqsA_Tetracycline plot (Figures 9a, 10a) reveals a distinct stable state, represented in dark blue, with the most stable state extracted at 65th ns and Gly279 forming a hydrogen bond interaction with the O group of Tetracycline at a distance of 1.81Å. The pqsA_559495 complex exhibited localized energy minima with only slight conformational changes. The most stable conformation was observed at 52nd ns; we analyzed the interaction patterns between the complexes. The key residues, such as Thr304, Tyr378, and Thr380, formed hydrogen bond interactions with the CID_559495; meanwhile, Asp382 developed a salt bridge interaction (Figures 9b, 10b). It was observed that the interactions identified during XP docking remained consistent after the MD simulation. This suggests that the complex maintained these crucial interactions throughout the simulation period. The pqsA_22217550 (Figures 9c, 10c) complex exhibited more flexibility and broader conformational states, with the most stable state identified at 74th ns. The key residues, such as Thr87, Asn84, and Thr164, were found to interact with CID_22217550 via hydrogen bond interaction, which demonstrated the stability and binding affinity of the complex. Overall, PCA/FEL analysis elucidated the dynamic behavior, conformational states, and significant interactions of the complexes. We have concluded that the identified lead phytocompounds could serve as a potent therapeutic choice for the deadliest diseases.

### 3.9 Molecular dynamics simulation of Protein Kinase CK2 with the phytocompounds

#### 3.9.1 Root mean square deviation (RMSD)

**FIGURE 11:**
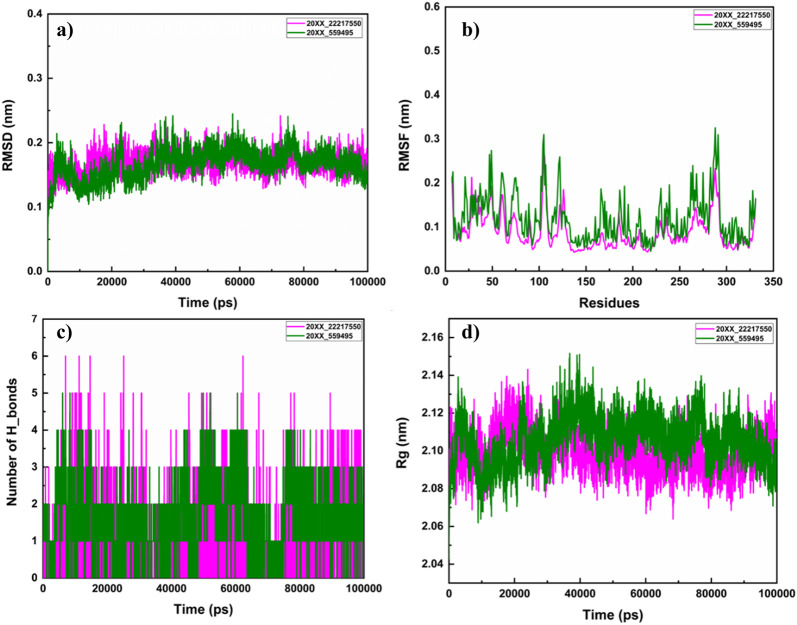
The analysis of MD simulations of the top CK2 complexes is illustrated, including **(a)** RMSD to evaluate structural stability, **(b)** RMSF for assessing residue flexibility, **(c)** profiles of hydrogen bonds to investigate intermolecular interactions, and **(d)** Rg to determine the overall compactness of the protein.

Figure 11 displays the MD simulation of the lead complexes, which includes RMSD, RMSF, hydrogen bond interactions, and Radius of gyration. The RMSD plot illustrates the structural stability of two lead complexes, 2OXX_22217550 and 2OXX_559495, for MD simulations. In this plot, the X-axis indicates the time measured in picoseconds (ps), which reflects the duration of the simulation. In this context, the Y-axis represents the RMSD in nanometers (nm), quantifying the deviation of atomic positions from their initial configuration. Following this initial period, both complexes reached an RMSD value of approximately 0.2 nm, signifying that the complexes achieved equilibrium and maintained structural stability throughout the simulation. The 2OXX_559495 complex exhibits smaller fluctuations in RMSD compared to the 2OXX_22217550 complex, indicating a slightly higher level of stability for 2OXX_559495. Overall, both complexes demonstrate commendable stability throughout the simulation, with 2OXX_559495 appearing to be marginally more stable. This level of stability is crucial for assessing the reliability of the ligand-protein complexes and their suitability for further investigation.

#### 3.9.2 Root mean square fluctuation (RMSF)

The RMSF analysis of 2OXX_22217550 and 2OXX_559495 provides valuable insights into the flexibility of individual protein residues throughout the MD simulation. Both complexes show low RMSF values for most residues, with fluctuations typically below 0.2 nm, indicating good structural stability. However, certain regions around residues 100, 250, and 300 exhibit higher RMSF values, suggesting localized flexibility. This increased flexibility is associated with loop regions. Notably, the 2OXX_559495 complex displays slightly higher peaks in these flexible regions compared to 2OXX_22217550, suggesting that it exhibits marginally greater flexibility in specific areas. In contrast, the core regions of the protein, particularly between residues 150 and 250, show minimal fluctuations for both complexes, reflecting a stable secondary structure, such as helices or sheets. These findings indicate that both complexes maintain structural integrity, with localized flexibility in functionally relevant regions. The system with 2OXX_22217550 appears to be slightly more stable overall, which may imply tighter ligand binding or reduced dynamic behavior compared to 2OXX_559495. These observations could have implications for the strength of the ligand’s interaction and the protein’s functional dynamics.

#### 3.9.3 Hydrogen bond interaction

The analysis of hydrogen bonds (H-bonds) from the MD simulations for two complexes provides important insights into the interaction dynamics between the ligands and the protein. Both complexes exhibit fluctuations in the number of H-bonds, reflecting the dynamic nature of ligand-protein interactions. Complex 2OXX_22217550 demonstrates a broader distribution of H-bond counts, with frequent peaks reaching 5 to 6 bonds. This suggests that 2OXX_22217550 engages in more frequent and stronger hydrogen bonding interactions, indicating a potentially more stable binding affinity. In contrast, complex 2OXX_559495 shows a more consistent pattern, with the majority of H-bond counts ranging between 1 and 3. This reflects a stable but relatively weaker interaction. The fluctuations observed in both complexes indicate moments of optimal binding geometry, where H-bond formation is maximized, as well as transient disruptions due to conformational changes in either the ligand or the protein. The higher peaks and overall greater H-bonding observed for 2OXX_22217550 suggest that it may form a more robust and energetically favorable complex with the protein compared to 2OXX_559495. These findings highlight the superior binding potential of 2OXX_22217550, making it a promising candidate for further development as a therapeutic agent.

#### 3.9.4 Radius of gyration (Rg)

The radius of gyration (Rg) is an important metric that quantifies the compactness of a protein-ligand complex and offers valuable insights into its structural stability. During the simulation, both systems demonstrated minor fluctuations in their Rg values, which remained confined within a narrow range, indicating consistent structural integrity. The 2OXX_22217550 complex exhibited slightly lower average Rg values in comparison to the 2OXX_559495 complex, suggesting that the protein-ligand interaction involving 2OXX_22217550 maintains a more compact and stable conformation. Conversely, the 2OXX_559495 complex displayed occasional increases in Rg, indicative of transient expansions within the complex. Nevertheless, these fluctuations were not substantial enough to signify instability in either system. The relatively stable Rg values observed for both complexes imply that the protein remains well-folded and structurally intact throughout the simulation period. The more compact structure identified for 2OXX_22217550 may suggest a stronger or tighter binding interaction, which could be associated with enhanced ligand efficacy. The observed low variations suggest that ligand interactions play a significant role in maintaining the protein’s stable, folded conformation. These findings support the potential bioactivity of the identified inhibitors, as compact and stable complexes are often associated with enhanced molecular recognition and functional efficacy. Meanwhile, the SASA plot illustrated the structural stability and folding of the docked complexes (Supplementary Figure S1b). These findings are consistent with the stability analyses and hydrogen bond assessments, thereby underscoring the potential of 2OXX_22217550 as a promising candidate for therapeutic development.

#### 3.9.5 MMPBSA

**FIGURE 12:**
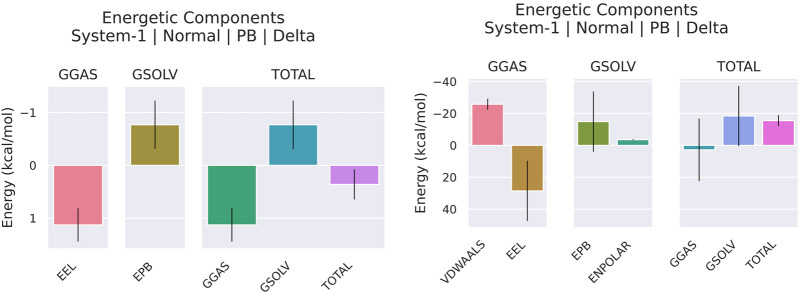
The analysis of binding free energy using MM/PBSA has been performed on the top lead complexes (CID_22217550_2OXX and CID_559495_2OXX). This analysis emphasizes the comparative binding affinities and the stability of the Protein_Ligand interactions.

Table 4; Figure 12 illustrate the binding free energy and its components for two lead complexes, 2OXX_22217550 (Table 4) and 2OXX_559495 (Table 4), evaluated using MMPBSA calculations. The analysis reveals significant differences in their interaction profiles. For 2OXX_22217550, the total binding free energy is slightly favorable at 0.36 kcal/mol. The electrostatic energy (EEL) and gas-phase energy (GGAS) contribute positively (1.13 kcal/mol each), but these are offset by unfavorable polar solvation energy (EPB, −0.77 kcal/mol) and solvation-free energy (GSOLV, −0.77 kcal/mol). The low standard deviations and standard errors of the mean (SEM) suggest a high level of stability in the calculations. Conversely, 2OXX_559495 demonstrates a much more favorable total binding free energy of −18.41 kcal/mol, primarily due to strong van der Waals interactions (VDWAALS, −25.76 kcal/mol) and moderate GGAS (−3.53 kcal/mol). Despite unfavorable contributions from electrostatic energy (EEL, 28.63 kcal/mol) and polar solvation energy (EPB, −14.87 kcal/mol), the strong van der Waals interactions dominate, leading to a stable binding complex. The higher SD and SEM indicate greater variability, possibly due to structural fluctuations.

**TABLE 4:** Binding affinity of lead complexes calculated through MMPBSA analysis.

Compounds	Frames	VDWAALS	EEL	EPB	ENPOLAR	GGAS	GSOLV	Total
20XX_22217550	Average	—	1.13	−0.77	—	1.13	−0.77	0.36
	SD	—	0.31	0.46	—	0.31	0.46	0.29
	SEM	—	0.01	0.01	—	0.01	0.01	0.01
20XX_559495	Average	−25.76	28.63	−14.87	−3.53	2.87	−18.41	−15.54
	SD	3.5	18.86	18.91	0.28	19.61	18.88	3.37
	SEM	0.11	0.6	0.6	0.01	0.62	0.6	0.11

The bar plots in Figure 12 visually summarize the energy contributions for each component. In summary, while 2OXX_22217550 features a near-neutral total binding energy, the strong van der Waals interactions in 2OXX_559495 indicate a more favorable complex, suggesting it is the more promising candidate for further exploration.

#### 3.9.6 PCA/FEL

**FIGURE 13:**
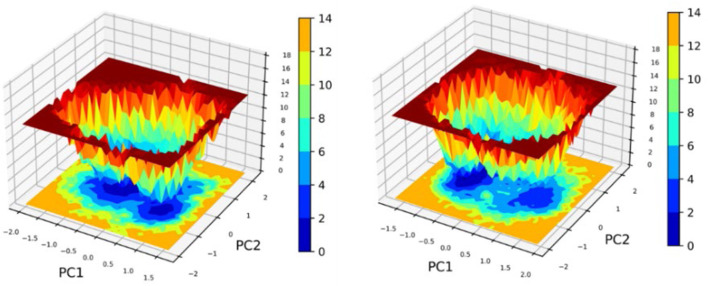
Principal Component Analysis (PCA) and Free Energy Landscape (FEL) analyses of the leading complexes (2OXX_22217550 and 2OXX_559495) represent the principal conformational dynamics and the energy minima that are associated with the stability of these complexes.

The Principal Component Analysis (PCA) and Free Energy Landscape (FEL) for two complexes are illustrated in Figure 13. These FEL plots effectively project the data onto the first two principal components (PC1 and PC2), facilitating the exploration of the conformational free energy landscape. The color gradient depicted in the plots represents the varying free energy levels, with dark blue indicating low-energy (most stable) regions and red signifying high-energy (less stable) regions. The landscape reveals a central minimum for the complex denoted as 2OXX_22217550, suggesting that the system primarily exhibits a stable conformation or an ensemble of similar conformations. In contrast, 2OXX_559495 exhibits a narrower and more sharply defined basin, implying that this complex is limited to a smaller set of energetically favorable conformations, thereby exhibiting reduced flexibility compared to 2OXX_22217550. This comparative analysis indicates that 2OXX_559495 may possess stronger conformational constraints or a more rigid structure, whereas 2OXX_22217550 likely reflects a more diverse conformational ensemble.

## 4 Discussion

Our previous study has demonstrated that the ethanol extract of ADEE exhibits significant antioxidant activity. The current *in silico* investigation supports these findings by revealing strong binding interactions between the phytocompounds in ADEE and Protein Kinase CK2. This kinase is a redox-sensitive signaling molecule involved in cancer progression and the regulation of cellular oxidative balance. Since CK2 is known to regulate survival pathways and is often overexpressed in TNBC cells (Kim et al., 2024; Firnau and Brieger, 2022), these identified interactions suggest that the anticancer effects of ADEE may be partially mediated by the modulation of intracellular oxidative stress. The observed dose-dependent cytotoxicity and apoptosis in MDA-MB-231 cells may be linked to the molecular antioxidant properties of the extract. This study investigates the therapeutic potential of ADEE, elucidating its efficacy against bacterial infections and breast cancer. The findings reveal that ADEE disrupts biofilm formation in *Pseudomonas aeruginosa* and induces apoptosis in MDA-MB-231 breast cancer cells. These results are consistent with prior research that highlights the diverse medicinal properties of *A. digitata*. Previous investigations have indicated that *A. digitata* fruit may aid in the management of diabetes through various mechanisms, including the modulation of glucose transport and insulin signaling pathways (Silva et al., 2023). This observation complements our findings regarding ADEE’s anticancer activity, as dysregulated glucose metabolism is frequently implicated in the development and progression of cancer. The methanol bark extract from *A. digitata* has demonstrated anti-cancer properties against MCF-7 breast cancer cells. The findings indicate that the extract exerts a dose-dependent cytotoxic effect, significantly inhibiting cell proliferation. Furthermore, the extract influences key apoptotic markers, specifically p53 and p21, suggesting a potential mechanism for its anti-tumor effects (Odunola, 2021). In comparison to other plant extracts evaluated for their effects on TNBC, the ethanol extract of *Aegle marmelos* demonstrated significant cytotoxic and pro-apoptotic effects within an effective range (Bhatti et al., 2013). The ethanolic extract of *Kigelia africana* displayed an IC_50_ of approximately 20 μg/mL (Kalsoom et al., 2024), while *Tabernaemontana catharinensis* extract had an IC_50_ of about 8.3 μg/mL in MDA-MB-231 cells (Mar et al., 2025). In contrast, *Ephedra alata Decne* ethanol extract exhibited a much lower effect, with an IC_50_ of around 364.6 μg/mL (Al Jaafreh, 2024). The results for ADEE are comparable to those of the more potent plant extracts, underscoring its potential as a viable treatment option for breast cancer. Moreover, the antioxidant activity of *A. digitata* fruit pulp, as demonstrated by Wasihun et al. (2023), supports our results concerning ADEE’s apoptotic effects, given that oxidative stress is a critical factor in the survival of cancer cells. Additionally, the analgesic and antihypertensive properties of *A. digitata* bark extract, as reported by Owoyele and Bakare (2018), Ntchapda et al. (2020), further emphasize its potential therapeutic benefits (Owoyele and Bakare, 2018; Ntchapda et al., 2020). The collective findings from both this study and existing literature suggest that ADEE may provide a multifaceted approach to disease management, targeting both infectious agents and cancer cells while potentially offering relief from pain and addressing cardiovascular issues. The antimicrobial activity of *A. digitata* bark extracts, highlighted by Masola et al. (2009), is congruent with our observations of ADEE’s ability to inhibit biofilm formation (Masola et al., 2009). These studies collectively underscore the potential of *A. digitata* bark as a source of antimicrobial agents, with implications for the treatment of bacterial infections, particularly those associated with biofilms. Furthermore, the reported antimalarial and antidepressant effects of *A. digitata* extracts (Shehu et al., 2020; Adeoye and Bewaji, 2018) further underscore the broad therapeutic potential of this plant. This study employed a combination of traditional molecular docking techniques and artificial intelligence-based validation to enhance the selection of lead compounds. Molecular docking conducted with Schrödinger Glide identified compounds 559495 and 22217550 as potential binders for the pqsA and CK2 targets, presenting docking scores ranging from −6.743 to −7.573 kcal/mol. To enhance confidence in the docking conformations, the complexes were assessed using GNINA, an artificial intelligence-powered docking platform that generated CNN-based scores and projected binding affinities. AI-driven discovery combines generative models with computational chemistry, progressing from empirical screening to rational design methodologies (Zeng et al., 2022). AI-driven technologies are transforming the drug development workflow. Techniques based on diffusion, such as DiffDock, are redefining the process of molecular docking. Additionally, deep neural networks exhibit exceptional accuracy in predicting binding affinities and facilitate large-scale virtual screening (Das, 2025). In this investigation, we employed GNINA, an AI-powered docking framework, to rescore and validate the docking results through XP docking. This integrated approach enhanced the reliability of pose selection and affinity estimation, leading to the identification of the most promising inhibitors from *A. digitata* against the selected targets. The outcomes of the *in vitro* cytotoxicity and apoptosis assays demonstrated a strong correlation with the *in silico* predictions. Compounds derived from *A. digitata*, which exhibited the highest binding affinities in the XP docking and GNINA rescoring analyses, along with stable structural behavior observed during molecular dynamics simulations, displayed the most distinguished cytotoxic and pro-apoptotic effects on TNBC cells. Furthermore, compounds that exhibited significantly favorable binding free energies in the MMPBSA study, as well as sustained hydrogen-bonding interactions within the target binding pocket, were found to induce substantial apoptotic effects *in vitro*. This highlights the critical significance of these molecular interactions. In conclusion, this study contributes to the expanding form of evidence supporting the medicinal properties of *A. digitata*. By demonstrating the dual-action efficacy of ADEE against bacterial biofilms and breast cancer, this study underscores its potential as a valuable resource for developing novel therapeutic strategies. Further research is warranted to thoroughly elucidate ADEE’s mechanisms of action and optimize its therapeutic applications, providing a robust foundation for future investigations into this multifaceted therapeutic agent.

## 5 Conclusion

This study provides compelling evidence that ADEE possesses the potential to address both bacterial infections and breast cancer. By demonstrating ADEE’s capacity to inhibit biofilm formation in *P. aeruginosa* and to induce apoptosis in MDA-MB-231 breast cancer cells, this research contributes significantly to the expanding body of literature that affirms the extensive therapeutic capabilities of *A. digitata*. Furthermore, this work reinforces previous findings regarding *A. digitata’s* properties, including its antioxidant, analgesic, antihypertensive, antibacterial, antimalarial, and antidepressant effects, thereby underscoring its potential as a promising source for new therapeutic compounds. Concurrently, it is significant to emphasize that these findings are preliminary. More extensive experimental studies and *in vivo* validation are required to demonstrate and enhance the therapeutic potential of ADEE. While this study provides a substantial basis and reinforces the concept that *A. digitata* could be a viable therapeutic agent, it should be considered as an initial phase that requires further investigation.

## Data Availability

The raw data supporting the conclusions of this article will be made available by the authors, without undue reservation.
