# Supplementary material for: Decoding the anticancer and biofilm-inhibiting efficacy of Adansonia digitata using experimental, AI-powered, and molecular modeling approaches
Source: Front Mol Biosci. 2025 Oct 29;12:1666360. doi: 10.3389/fmolb.2025.1666360 (PMC12605543; doi:10.3389/fmolb.2025.1666360)
Supplement: Supplementary file 1 [file Supplementaryfile1.docx]

**Supplementary Figure 1: Solvent Accessible Surface Area (SASA) calculation of the Phytocompounds_target complexes**

1. **SASA plot of pqsA_complexes determined their stability and folding**

**

**

1. **SASA plot of CK2_complexes illustrated the structural stability and folding**

**

**
